# Towards Wearable Comprehensive Capture and Analysis of Skeletal Muscle Activity during Human Locomotion

**DOI:** 10.3390/s19010195

**Published:** 2019-01-07

**Authors:** Christina Zong-Hao Ma, Yan To Ling, Queenie Tsung Kwan Shea, Li-Ke Wang, Xiao-Yun Wang, Yong-Ping Zheng

**Affiliations:** 1 Department of Biomedical Engineering, The Hong Kong Polytechnic University, Hong Kong, China; christina.ma@connect.polyu.hk (C.Z.-H.M.); jane.yt.ling@connect.polyu.hk (Y.T.L.); queenie.tk.shea@connect.polyu.hk (Q.T.K.S.); akewanglike@hotmail.com (L.-K.W.); 2 Department of Rehabilitation, Jönköping University, 551 11 Jönköping, Sweden; 3 Guangdong Work Injury Rehabilitation Center, Guangzhou 510440, China; xiaoyunwang77@hotmail.com

**Keywords:** motion capture and analysis, wearable, ultrasound imaging, electromyography (EMG), sonomyograph (SMG), mechanomyography (MMG), plantar force, joint angle

## Abstract

Background: Motion capture and analyzing systems are essential for understanding locomotion. However, the existing devices are too cumbersome and can be used indoors only. A newly-developed wearable motion capture and measurement system with multiple sensors and ultrasound imaging was introduced in this study. Methods: In ten healthy participants, the changes in muscle area and activity of gastrocnemius, plantarflexion and dorsiflexion of right leg during walking were evaluated by the developed system and the Vicon system. The existence of significant changes in a gait cycle, comparison of the ankle kinetic data captured by the developed system and the Vicon system, and test-retest reliability (evaluated by the intraclass correlation coefficient, ICC) in each channel’s data captured by the developed system were examined. Results: Moderate to good test-retest reliability of various channels of the developed system (0.512 ≤ ICC ≤ 0.988, *p* < 0.05), significantly high correlation between the developed system and Vicon system in ankle joint angles (0.638R ≤ 0.707, *p* < 0.05), and significant changes in muscle activity of gastrocnemius during a gait cycle (*p* < 0.05) were found. Conclusion: A newly developed wearable motion capture and measurement system with ultrasound imaging that can accurately capture the motion of one leg was evaluated in this study, which paves the way towards real-time comprehensive evaluation of muscles and joint motions during different activities in both indoor and outdoor environments.

## 1. Introduction

Human posture control and motion require sufficient coordination of central nervous and musculoskeletal systems. In the musculoskeletal system, the ankle plantar-flexors generate necessary force for foot propulsion during walking [1] and running [2], as well as maintaining the static and dynamic balance control [3] in human. The function of plantar-flexor has been suggested as one strong indicator of gait performance [4]. Weakness [1] and contracture [5] of plantar-flexor tend to adversely affected balance and gait, leading to higher risk of falls [6].

Various measurement modalities have been used to evaluate the functions of plantar-flexors, including surface electromyography (EMG) measuring muscle electrical activity [7], goniometer measuring joint ankles [7], surface mechanomyography (MMG) measuring muscle vibration [8], infrared motion capture and analyzing system with cameras and reflective markers measuring kinematics and kinetics [5], floor-mounted force plates measuring ground reaction force [5], and ultrasound imaging evaluating changes in muscle structures [2,9,10,11,12]. However, such measurement modalities need to be wired to computers for data collection and analysis. This restrained their application to indoor laboratories only. The traditional large ultrasound probe also makes the available body regions and motions for assessment/measurement limited. The indoor infrared motion capture and analyzing system, floor-mounted force plates, and bulky ultrasound imaging systems are generally expensive, and require a specific space/laboratory to install such instruments. All these factors narrowed the application of the above-mentioned measurement modalities into a limited number of research groups and hospitals only.

Research and development of wearable devices have been gaining more attention and efforts recently. Attempts have been made to develop smart wearable devices that allow human motion capture [13,14,15,16,17] and estimation of biometric properties [18,19,20], and even provide corresponding biofeedback to users based on the analysis [21,22] with various sensors and feedback units. Improvements of balance and gait control have been achieved with such devices [23,24,25,26,27]. However, each of these systems only measures one aspect of human motion, leading to a non-comprehensive analysis when using a single system. To achieve a comprehensive analysis, connection, and synchronization of multiple motion capture systems are required during the experiment and evaluation.

With the current state-of-the-art technology, various sensors and measurement units can be integrated together to develop some wearable systems enabling comprehensive human motion capture analysis. Sonomyograph (SMG) is a non-invasive approach of ultrasound imaging that has been widely applied for measuring the human muscle-tendon complex [28,29,30,31]. However, due to the technology limitations, previous SMG systems needed cables to connect to computers for data transmission and analysis. Thin-film force sensors could capture the plantar force information accurately and reliable [32]. Goniometers [28], surface EMG [28,29], and surface MMG [8] can capture the real-time joint angles and muscle activity in vivo. To the knowledge of the authors, so far, none of the previous studies have attempted to develop a wearable human motion analyzing system providing the real-time ultrasound imaging of SMG, surface MMG, surface EMG, joint angles, and plantar force distribution that enables comprehensive analysis of human motion in both indoor and outdoor conditions.

This project aimed to (1) introduce a newly developed wearable mobile sensing system with wirelessly transmitted real-time ultrasound imaging for human motion analysis (named as ‘mobile SMG system’); and (2) investigate the feasibility and reliability of the proposed wearable device on accurate capture and analysis of the motion of one leg during gait in healthy young subjects.

## 2. Materials and Methods

### 2.1. Subjects

A convenience sampling approach was adopted to recruit 10 healthy young subjects aged between 18 and 35 years. Subjects should be in a healthy state with no need of assistive devices for ambulation. Subjects with previous history of lower-limb injury or foot deformity were excluded from this study.

Ethical approval was granted from the authority of the Human Subjects Ethics Sub-committee of The Hong Kong Polytechnic University (HSEARS20180418003). All participants signed an informed consent form after receiving oral and written descriptions of the research and the experimental procedures prior to the experiments.

### 2.2. Wearable Motion Capture and Analysis System with Real-Time SMG (Mobile SMG System)

The system consisted of an ultrasound probe (Bandwidth 7.5 MHz ± 35%), two sets of surface EMG electrodes (272-Bx, Noraxon USA Inc., Scottsdale, AZ, USA), three thin-film force sensors (A301, Tekscan Co., Ltd., South Boston, MA, USA), a MMG sensor (N1000060, VTI Technologies Oy, Vantaa, Finland), a two-axis goniometer (model XM110, Penny and Giles Biometrics Ltd., Gwent, UK), a wireless Wi-Fi transmitter module (802.11.n), and two rechargeable lithium batteries (SNP-4200, D.seven Co., Ltd., Shenzhen, China). It was capable of providing the real-time B-mode ultrasound image of muscles, and measuring the muscle activation, muscle vibration, foot plantar force, and joint angle simultaneously. The ultrasound probe and sensors were wirelessly connected to a laptop, which collected and analyzed data. The frame rate of ultrasound images was 10 Hz, and the sampling frequency of the other sensors was 5 kHz. The multiple sensors were synchronized by a system clock from the field-programmable gate array (FPGA). The high-performance FPGA was used to control the data acquisition, conversion, and transfer of all the channels and ultrasound image.

**Figure 1:**
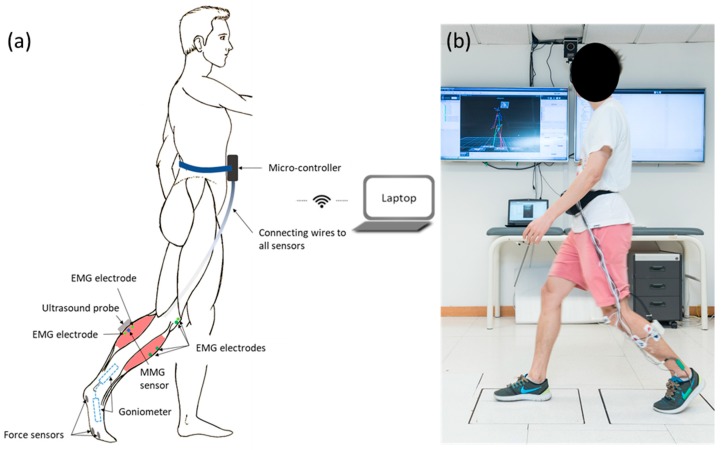
(**a**) An illustration of the wearable mobile sensing system with real-time ultrasound imaging and location of ultrasound probe, electromyography (EMG) electrode, mechanomyography (MMG) electrode, force sensors, and goniometer at shank and foot. (**b**) A demonstration of a subject wearing the wearable mobile sensing system.

As shown in Figure 1a, an ultrasound probe, a MMG sensor, and one set of EMG electrodes were put along the direction of muscle fiber of lateral head of gastrocnemius [7]. They were used to capture the real-time B-mode ultrasound image, the vibration motion on the skin surface generated by the muscle activity and the electrical activities of the muscle of lateral head of gastrocnemius, respectively. The other set of EMG electrodes were put along the direction of the muscle fiber of the tibialis anterior. The reference electrodes of both EMG channels were put on the bony prominence of patella. One end of goniometer was put at the medial foot along the direction of the first array, and the other end of goniometer was put at the medial shank along the direction of shank, to capture the ankle joint angle. The three force sensors were put at the plantar surface of the first metatarsal head, the second metatarsal head, and the heel, by a certified orthotist, to capture the force at plantar surface of the foot. The force sensors were attached to the right-side insole of a pair of flat insoles (2 mm thickness) manufactured using medium firm (30–35 Shore A hardness) ethylene-vinyl acetate (EVA, Foot Specialist Footcare & Products Co. Ltd., Hong Kong, China) by a CERTIFIED ORTHOTIST (Figure 1a). Figure 1b demonstrates how the sensors and the probe were attached to the subject. Note that during the experiments, the sensors were attached to the right leg instead of the left leg, and the goniometer was attached to the medial shank instead of the lateral shank.

### 2.3. Experimental Procedure and Data Acquisition.

This study was conducted in a university locomotion laboratory. After explaining to the subjects about the experimental design, all the sensors were attached to each subject’s right foot and shank (Figure 1). Then each subject was asked to walk along a 7-m long level walkway with a comfortable speed for three walking trials consecutively.

The plantar-flexor muscle activity during walking was evaluated by the ultrasound image, MMG, and EMG signals of the mobile SMG system. The plantarflexion and dorsiflexion motion during walking was assessed by the joint angle measured by the goniometer, and the plantar force measured by the force sensors. The muscle area of plantar-flexor was measured from the ultrasound image, and was used to calculate the changes during walking.

An eight-camera three-dimensional (3D) motion capture system with good accuracy and reliability [33,34] (Vicon Nexus 2.5.1, Vicon NexusTM, Vicon Motion Systems Ltd., Yarnton, UK) sampling at 250 Hz and two floor-mounted force plates (OR6, Advanced Mechanical Technology, Inc., Watertown, MA, USA) sampling at 1000 Hz, was used to measure the 3D kinematic and kinetic data, specifically the plantar force and ankle joint angle, of the right side in subjects during walking. A built-in lower limb marker set (plug-in gait model) was adopted, in which 16 infrared reflective markers were affixed to both sides at the heels, foot dorsum, lateral malleolus, lateral femoral condyles, middle of thighs/shanks, anterior superior iliac spines, and posterior superior iliac spines. The kinematic and kinetic data were measured and analyzed using the plug-in gait model in the Vicon system.

### 2.4. Signal Processing

**Figure 2:**
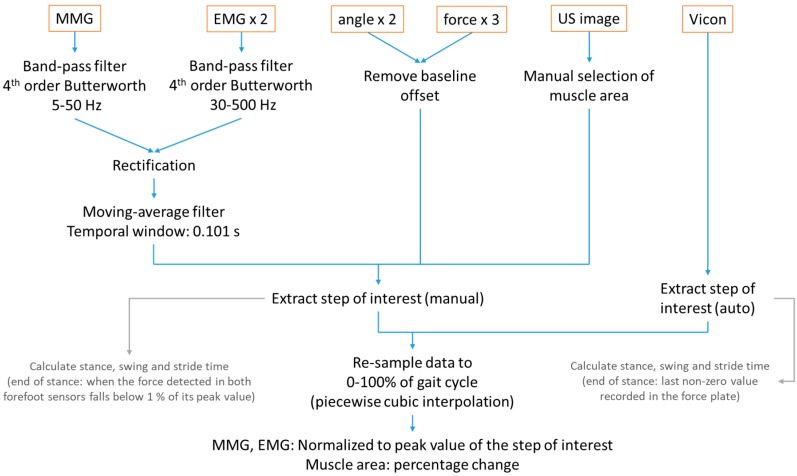
Flow chart for data processing and analysis.

**Figure 3:**
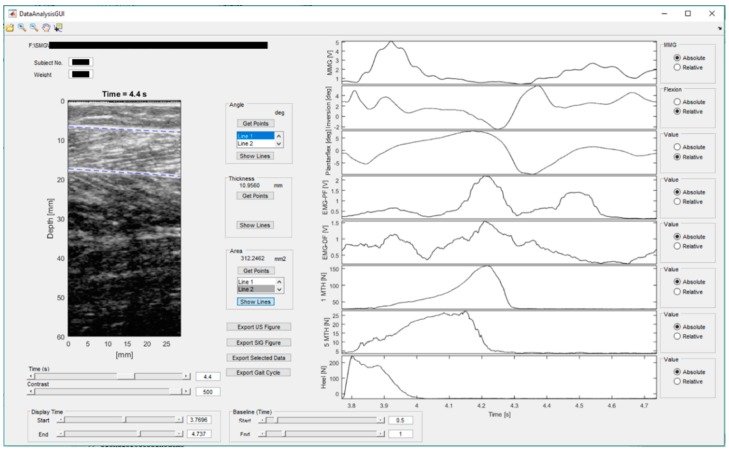
An example of user-interface (UI) of the mobiles SMG system.

The data were analyzed using the Matlab (version 2016b, The MathWorks Inc., Natick, MA, USA). A flow chart for the process is included in Figure 2. An example showing the user-interface (UI) of the mobiles SMG system is illustrated in Figure 3.

MMG and EMG data was filtered using the 4th-order Butterworth band-pass filter of 5–50 Hz and 30–500 Hz, respectively. The signals were then rectified and filtered with a moving-average filter of temporal window of 0.101 s [35]. Baseline offsets of the recorded angles and forces were removed with subtractions of the steady-state baseline values. In the ultrasound images, muscle boundaries of the gastrocnemius muscle were indicated manually by drawing two lines in each frame by a trained practitioner (Figure 3). The muscle area was then calculated from the area between the two lines in each frame. The measured muscle area at heel strike was set as the baseline to calculate the changes in muscle area in a gait cycle. The step of interest was extracted manually from the time-series data with reference to the plantar forces. In calculation of stance and swing time, the end of stance phase is regarded as the time when the force detected fell below 1% of their peak values. The data was then resampled to 0–100% of gait cycle using piecewise cubic interpolation. EMG and MMG signals were normalized to their peak values during walking [36]. Percentage change of muscle area with respect to the beginning of a gait cycle was calculated. The forces measured at the first metatarsal head, the second metatarsal head, and the heel were summed to calculate the plantar force of full foot, and normalized by dividing by each subject’s body mass.

Vicon data was extracted to the step of interest with reference to the foot progress angle in the sagittal plane. In calculation of stance and swing time, the end of stance phase is regarded as the time when the force detected by the force plate returned to zero. The step was then resampled to 0–100% of gait cycle using piecewise cubic interpolation. Therefore, the step data from two systems were synchronized by the defined gait cycle.

The plantar force data captured by the force sensors in the wearable mobile sensing system were compared to the plantar force captured by the floor-mounted force plate in the Vicon system. The ankle joint angles (plantarflexion/dorsiflexion and inversion/eversion) captured by the goniometers in the wearable mobile sensing system were compared to the corresponding joint angle captured by the Vicon system.

### 2.5. Statistical Analysis

Statistical analysis was performed using Statistical Package for Social Sciences (SPSS, version 25.0, IBM Corporation, Armonk, NY, USA). Each gait cycle was divided into 21 time points from 0% to 100% (5% interval) for statistical analysis. **Paired *t*-test** was performed to examine the existence of significant change between two consecutive time points in each channel of the data captured by the mobile SMG system, to identify the statistically significant features of muscle activity and joint motion in a gait cycle. **Pearson correlation test** was performed to compare the ankle kinetic data captured by the mobile SMG system and the Vicon system, to examine the validity of the mobile SMG system. **Intraclass correlation coefficient (ICC) test** was performed to evaluate the test-retest reliability in each channel of the data captured by the mobile SMG system. The level of significance was set at 0.05.

## 3. Results

A total of 10 healthy subjects participated in this study (seven males and three females, aged 26.4 ± 2.4 years, height 169.8 ± 12.2 cm, and weight 65.1 ± 14.9 kg).

**Figure 4:**
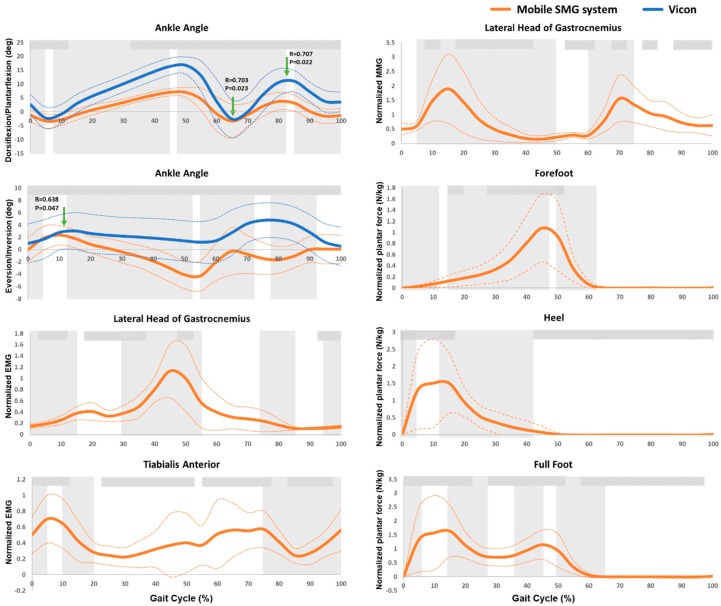
Ankle activities in a gait cycle measured by Vicon and the newly developed mobile SMG system of ten healthy subjects. P: P value; R: Pearson’s correlation coefficient; 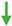: significantly high correlation between the Vicon and mobile SMG systems; 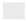: significant changes in trend in consecutive 5% intervals; 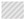: significantly high correlation in intraclass correlation coefficient (ICC) among three trials; The bolded orange line illustrates the averaged data measured by the mobile SMG system; The bolded blue line illustrates the averaged data measured by the Vicon system; The thin dashed line illustrates the standard deviation (SD) of each corresponding bold line.

The measured ankle activities in a gait cycle measured by the newly developed mobile SMG system and the Vicon system of 10 healthy subjects were illustrated in Figure 4. The trend of ankle plantar-/dorsi-flexion and in-/e-version measured by the mobile SMG device was similar to that measured by the Vicon system. Significantly high correlation between the goniometer of the introduced mobile SMG system and the Vicon system was found in peak plantarflexion (R = 0.703, *p* = 0.023) and peak dorsiflexion (R = 0.707, *p* = 0.022) during swing phase, as well as the peak eversion (R = 0.638, *p* = 0.047) during initial stance phase.

As shown in Figure 4, the intensity of MMG signal increased since heel-strike and reached a peak during loading response (1.90 ± 1.19, *p* < 0.05), followed by a decrease until reaching a plateau during terminal stance phase. The intensity of MMG signal increased again and reached a second peak during pre-swing phase (1.56 ± 0.80, *p* < 0.05), followed by another decrease until the end of a gait cycle.

The intensity of EMG signal at gastrocnemius increased and reached a peak during loading response phase (0.41 ± 0.16, *p* < 0.05). It then decreased and increased again, reaching a second peak during terminal stance phase (1.13 ± 0.46, *p* < 0.05). The intensity of EMG signal at tibialis anterior increased first and reached a peak during loading response significantly (0.70 ± 0.30, *p* < 0.05), followed by a decrease during mid-stance phase. Such EMG signal then increased in the following gait cycle, and decreased again since initial swing phase, reaching a minimal EMG signal intensity during mid-swing phase (Figure 4).

As shown in Figure 4, the normalized plantar force of full foot increased significantly since the initial contact and reached a peak during loading response (1.64 ± 1.27 N/kg, *p* < 0.05). It then decreased significantly and reached a trough during mid-stance phase, followed by a significant increase that reached a second peak during terminal stance phase (1.15 ± 0.38 N/kg, *p* < 0.05). Such plantar force then decreased to 0 during pre-swing phase of a gait cycle. The first peak normalized plantar force of full foot was contributed by the peak plantar force of heel, and the second one was contributed by the peak plantar force of forefoot in a gait cycle (Figure 4).

**Figure 5:**
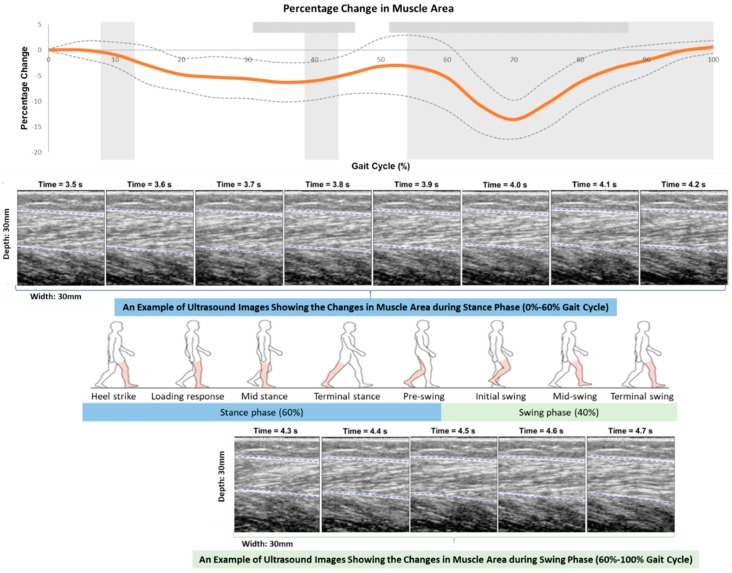
Percentage changes in muscle area in a gait cycle measured by the newly developed mobile SMG system of ten healthy subjects. 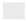: significant changes in trend in consecutive 5% intervals; 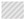: significantly high correlation in the intraclass correlation coefficient (ICC) among three trials.

As shown in Figure 5, the gastrocnemius muscle area kept being below the baseline muscle area during the whole gait cycle. It firstly reduced since heel-strike, and reached a trough (6.28% ± 3.85%, *p* = 0.001) in mid-stance, followed by an increase until the terminal-stance phase. This muscle area reduced again from the pre-swing phase and reached a minimal muscle area (13.6% ± 3.8%, *p* = 0.002) during initial swing phase, followed by an increase until the terminal-swing phase in subjects. The minimal muscle area of a gait cycle was found in initial swing phase, which was 142.4% less than the trough in mid-stance phase (*p* < 0.001).

Table 1 and Figure 4 summarize the ICC test results examining the test-retest reliability of the integrated sensors of the mobile SMG device during a gait cycle, including the ultrasound probe, MMG sensor, goniometer, EMG electrodes, and force sensors. Generally, the test-retest reliability of the various sensors of the mobile SMG device ranged from moderate to good. Specifically, the averaged significant ICC values (<0.05) suggested that there was moderate test-retest reliability in the measured changes in muscle area (by ultrasound probe, ICC = 0.552), muscle vibration (by MMG sensor, ICC = 0.653), ankle in-/e-version (by goniometer, ICC = 0.746), electrical activity of gastrocnemius (by EMG electrode, ICC = 0.745); and good test-retest reliability in electrical activity of tibialis anterior (by EMG electrode, ICC = 0.773), ankle plantar-/dorsi-flexion (by goniometer, ICC = 0.782), and plantar force of full foot (by force sensors, ICC = 0.781) during a gait cycle.

## 4. Discussion

A novel wearable mobile sensing system with real-time ultrasound imaging for human motion capture and analysis was developed and applied for gait analysis in this study. In addition to supporting the moderate to good test-retest reliability of this system, the findings of this study also unveiled some features of muscle activity, especially the gastrocnemius, during walking upon a comprehensive data capture and analysis.

Moderate to good test-retest reliability of various measurement channels of the mobile SMG system and strong correlation between this system with the Vicon system regarding the ankle plantar-/dorsi-flexion angle measurement was found. The averaged ICC values of EMG, goniometer, and force sensors were found to be 0.75 or above, suggesting good test-retest reliability in these measurement channels. Meanwhile, moderate test-retest reliability was found in MMG and ultrasound imaging (0.5 < ICC < 0.75, *p* < 0.05). The ankle plantar-/dorsi-flexion angle measured by the mobile SMG device was comparable to that of the Vicon system, and [35,37] strong correlation in peak dorsiflexion and peak plantarflexion angle during swing phase was found between the two systems (R > 0.5, *p* < 0.05). The ankle inversion/eversion angle measured by the mobile SMG device was not quite comparable to that of the Vicon system. One possible reason could be due to the different axis of rotation in angle measurement, i.e., Vicon system estimated the angle from the built-in model, while the goniometer measured the angle from the medial side of the ankle joints.

The trends of the EMG [35,37], MMG [37], muscle area [12], plantar force [38,39], and ankle angle [38,39] were comparable to previous studies. A large peak during loading response and a second peak during initial swing in MMG signal were found in this study. This is in accordance with a previous study identifying a similar trend in MMG signal during walking at slow walking speed [37]. Similar to previous studies, a large peak during terminal stance in EMG signal of the gastrocnemius was also found in this study [35,37].

While previous studies reported that the time lag between the maximal EMG and MMG signal was larger than the electromechanical delay in gastrocnemius [35], this study further identified that the time lag between the EMG signal, joint angle, and reduction of muscle area were also larger than the electromechanical delay in gastrocnemius during walking. A time lag was found between the peak of EMG signal (approximately 45% of gait cycle) and other signals of ankle plantarflexion (approximately 65% of gait cycle), MMG signal (approximately 70% of gait cycle), and maximal reduction of muscle area (approximately 70% of gait cycle). However, due to the relatively low frame rate of the ultrasound probe in this study, future studies adopting faster imaging modalities of muscles are needed to verify this.

Co-activation of the gastrocnemius and tibialis anterior was observed during most time of a gait cycle by the newly developed mobile SMG system used in this study. This was supported by the EMG signals of the two muscles, and was also similar to the findings of previous studies [35,40].

The muscle area of lateral head of gastrocnemius muscle was found to decrease during the swing phase and reach a trough during mid-swing phase in a gait cycle. This finding is in accordance previous studies investigating the changes of muscle fascicle length of the gastrocnemius muscle, which also identified that the muscle fascicle length decreased and reached a trough during swing phase [11,12].

A mobile motion analysis system with real-time ultrasound imaging would enable the comprehensive investigation of real-time muscle’s behavior and adaption during different activities in vivo in humans. The success of this project will pave the way to real-time in-viva analysis of muscle activity in both indoor and outdoor environments, which offers tremendous potential health benefits. This device can be widely applied to evaluate the human posture and motion, as well as the treatment outcomes of patients in the future. It is a systematic extension of our previous research, which utilizes the team’s unique expertise in ultrasound imaging technology, as well as posture and gait analysis in various populations.

This study has several limitations. The sample size of this study is relatively small. The frame rate of the current prototype was only 10 Hz. Future efforts could be put on optimizing this protype by increasing the frequency and capacity. The ultrasound image quality also can be further improved so as to produce image with better quality and facilitate more complexed image processing. The current prototype still needed wires to connect multiple sensors. This could be improved with flexible, conformable devices that are tailored to make such measurements in a way that is mechanically invisible in the future. This study has used the peak value during a walking trial as the reference value for normalization of the EMG and MMG signal [36]. Future studies may consider using other reference values, such as the peak value during maximum voluntary contraction (MVC) through the manual muscle testing (MMT), and compare if any differences in results existed between these methods.

## 5. Conclusions

This study introduced a newly developed wearable motion capture and measurement system with ultrasound imaging (mobile SMG system), evaluated its reliability and validity, and applied it to monitor muscle activities and joint motion in a gait cycle. Moderate to good test-retest reliability of the various channels of this system was found. This study also identified some interesting changes in lateral head of gastrocnemius during a gait cycle by comprehensively evaluating the muscle activity with the multiple measurement modalities integrated in the mobile SMG system. This paves the way towards real-time comprehensive evaluation of muscles during different activities in both indoor and outdoor environments. It is also expected that such kind of wearable motion capture and measurement system can be used in clinical field. Some potential applications could be diagnosis of skeletal muscle problems, objective evaluation of musculoskeletal disorders, and assessment of balance and gait control before and after rehabilitation trainings or orthotic management.

## Figures and Tables

**Table 1:** Statistical results of reproducibility of the mobile SMG device during a gait cycle of ten healthy subjects.

Gait Cycle	% Muscle Area		MMG		In-/E-Version		Plantar-/Dorsi-Flexion		EMG_ Plantarflexor		EMG_ Dorsiflexor		Plantar Force	
%	ICC	*p* Value	ICC	*p* Value	ICC	*p* Value	ICC	*p* Value	ICC	*p* Value	ICC	*p* Value	ICC	*p* Value
0	NA	NA	−0.017	0.484	0.761	0.003	0.647	0.013	0.427	0.074	0.712	0.006	NA	NA
5	0.041	0.415	0.321	0.134	0.711	0.006	0.524	0.038	0.662	0.011	0.84	0.001	0.919	<0.001
10	−0.496	0.983	0.803	0.001	0.655	0.011	0.522	0.038	0.754	0.003	0.623	0.016	0.988	<0.001
15	0.195	0.221	0.456	0.061	0.709	0.006	0.432	0.072	0.408	0.083	0.347	0.117	0.913	<0.001
20	−0.243	0.747	0.81	0.001	0.561	0.028	0.258	0.181	0.744	0.004	0.386	0.094	0.524	0.038
25	−0.448	0.999	0.667	0.01	0.501	0.045	0.4	0.087	0.831	0.001	0.889	<0.001	0.471	0.056
30	−0.249	0.806	0.589	0.022	0.54	0.033	0.487	0.05	0.877	<0.001	0.695	0.007	0.768	0.002
35	0.512	0.027	0.731	0.004	0.653	0.012	0.621	0.016	0.978	<0.001	0.737	0.004	0.915	<0.001
40	0.521	0.041	0.529	0.036	0.828	0.001	0.775	0.002	0.413	0.081	0.592	0.021	0.799	0.001
45	0.499	0.042	0.316	0.138	0.885	<0.001	0.741	0.004	−0.204	0.742	0.816	0.001	0.803	0.001
50	0.378	0.087	0.359	0.11	0.868	<0.001	0.548	0.031	0.705	0.006	0.79	0.002	0.766	0.003
55	0.607	0.016	0.593	0.021	0.857	<0.001	0.734	0.004	−0.105	0.605	0.448	0.065	0.105	0.331
60	0.644	0.018	0.547	0.032	0.909	<0.001	0.95	<0.001	−0.018	0.485	0.943	<0.001	0.646	0.013
65	0.416	0.045	0.01	0.448	0.914	<0.001	0.914	<0.001	0.353	0.113	0.872	<0.001	0.813	0.001
70	0.421	0.049	0.615	0.017	0.88	<0.001	0.929	<0.001	0.075	0.366	0.744	0.004	0.825	0.001
75	0.658	0.008	0.05	0.397	0.926	<0.001	0.938	<0.001	0.001	0.459	0.919	<0.001	0.521	0.039
80	0.688	0.009	0.589	0.022	0.898	<0.001	0.933	<0.001	−0.032	0.504	0.263	0.177	0.714	0.006
85	0.306	0.169	−0.072	0.559	0.821	0.001	0.898	<0.001	0.019	0.436	0.685	0.008	0.717	0.005
90	0.317	0.147	0.554	0.03	0.803	0.001	0.743	0.004	0.266	0.175	0.844	0.001	0.8	0.001
95	0.048	0.399	0.658	0.011	0.853	<0.001	0.593	0.021	0.569	0.026	0.659	0.011	0.843	0.001
100	−0.253	0.835	0.799	0.001	0.88	<0.001	0.665	0.01	0.583	0.023	0.463	0.059	0.461	0.059
**Sig. ICC (mean ± SD)**	**0.552 ± 0.101**		**0.653 ± 0.102**		**0.782 ± 0.132**		**0.746 ± 0.157**		**0.745 ± 0.135**		**0.773 ± 0.107**		**0.781 ± 0.129**	

Sig. ICC: Significant difference in intraclass correlation coefficient (ICC); SD: standard deviation; The highlighted cells indicate significant difference existed in ICC test (*p* < 0.05).

## References

[1] Davies B.L., Hoffman R.M., Healey K., Zabad R., Kurz M.J. (2017). Errors in the ankle plantarflexor force production are related to the gait deficits of individuals with multiple sclerosis. Hum. Move. Sci..

[2] Lai A.K., Lichtwark G.A., Schache A.G., Pandy M.G. (2018). Differences in in-vivo muscle fascicle and tendinous tissue behaviour between the ankle plantarflexors during running. Scand. J. Med. Sci. Sports.

[3] Norris E.S., Wallmann H.W. (2016). Static and dynamic balance after ankle plantarflexor fatigue in older adults. Phys. Occup. Ther. Geriatr..

[4] Mañago M.M., Hebert J.R., Kittelson J., Schenkman M. (2018). Contributions of Ankle, Knee, Hip, and Trunk Muscle Function to Gait Performance in People with Multiple Sclerosis: A Cross-Sectional Analysis. Phys. Ther..

[5] Gaudreault N., Gravel D., Nadeau S. (2009). Evaluation of plantar flexion contracture contribution during the gait of children with Duchenne muscular dystrophy. J. Electromyogr. Kinesiol..

[6] Deandrea S., Lucenteforte E., Bravi F., Foschi R., La Vecchia C., Negri E. (2010). Risk factors for falls in community-dwelling older people: A systematic review and meta-analysis. Epidemiology.

[7] Fujisawa H., Suzuki H., Nishiyama T., Suzuki M. (2015). Comparison of ankle plantar flexor activity between double-leg heel raise and walking. J. Phys. Ther. Sci..

[8] Beck T.W., Housh T.J., Johnson G.O., Cramer J.T., Weir J.P., Coburn J.W., Malek M.H. (2007). Does the frequency content of the surface mechanomyographic signal reflect motor unit firing rates? A brief review. J. Electromyogr. Kinesiol..

[9] Dick T.J., Nuckols R.W., Sawicki G.S. Tuned or not? Ultrasound measurements of soleus fascicle dynamics during human walking with elastic ankle exoskeletons. Proceedings of the 41st Annual Meeting of the American Society of Biomechanics.

[10] Miyoshi T., Takagi M., Yimit A., Hagihara Y., Komeda T. Task-Dependent Gastrocnemius Fiber Movement in Humans while Standing as Revealed by Ultrasound Images. Proceedings of the ICSSE.

[11] Aggeloussis N., Giannakou E., Albracht K., Arampatzis A. (2010). Reproducibility of fascicle length and pennation angle of gastrocnemius medialis in human gait in vivo. Gait Posture.

[12] Lichtwark G., Bougoulias K., Wilson A. (2007). Muscle fascicle and series elastic element length changes along the length of the human gastrocnemius during walking and running. J. Biomech..

[13] Ramanathan A., Kiran P., Arnold G., Wang W., Abboud R. (2010). Repeatability of the Pedar-X® in-shoe pressure measuring system. Foot Ankle Surg..

[14] Slyper R., Hodgins J.K. Action capture with accelerometers. Proceedings of the 2008 ACM SIGGRAPH/Eurographics Symposium on Computer Animation.

[15] Riaz Q., Tao G., Krüger B., Weber A. (2015). Motion reconstruction using very few accelerometers and ground contacts. Graph. Models.

[16] Vlasic D., Adelsberger R., Vannucci G., Barnwell J., Gross M., Matusik W., Popović J. Practical motion capture in everyday surroundings. Proceedings of the ACM Transactions on Graphics (TOG).

[17] Stollenwerk K., Müllers J., Müller J., Hinkenjann A., Krüger B. Evaluating an Accelerometer-based System for Spine Shape Monitoring. Proceedings of the Computational Science and Its Applications—ICCSA.

[18] Riaz Q., Vögele A., Krüger B., Weber A. (2015). One small step for a man: Estimation of gender, age and height from recordings of one step by a single inertial sensor. Sensors.

[19] Kobsar D., Ferber R. (2018). Wearable Sensor Data to Track Subject-Specific Movement Patterns Related to Clinical Outcomes Using a Machine Learning Approach. Sensors.

[20] Fröhlich H., Claes K., De Wolf C., Van Damme X., Michel A. (2018). A Machine Learning Approach to Automated Gait Analysis for the Noldus Catwalk System. IEEE Trans. Biomed. Eng..

[21] Ma C.Z.-H., Wong D.W.-C., Lam W.K., Wan A.H.-P., Lee W.C.-C. (2016). Balance improvement effects of biofeedback systems with state-of-the-art wearable sensors: A systematic review. Sensors.

[22] Wan A.H., Wong D.W., Ma C.Z., Zhang M., Lee W.C. (2016). Wearable vibrotactile biofeedback device allowing identification of different floor conditions for lower-limb amputees. Arch. Phys. Med. Rehabil..

[23] Ma C.Z.-H., Zheng Y.-P., Lee W.C.-C. (2018). Changes in gait and plantar foot loading upon using vibrotactile wearable biofeedback system in patients with stroke. Top. Stroke Rehabil..

[24] Ma C.Z.-H., Wan A.H.-P., Wong D.W.-C., Zheng Y.-P., Lee W.C.-C. (2015). A Vibrotactile and Plantar Force Measurement-Based Biofeedback System: Paving the Way towards Wearable Balance-Improving Devices. Sensors.

[25] Ma C.Z.-H., Lee W.C.-C. (2017). A wearable vibrotactile biofeedback system improves balance control of healthy young adults following perturbations from quiet stance. Hum. Move. Sci..

[26] Sienko K.H., Balkwill M.D., Oddsson L.I., Wall C. (2013). The effect of vibrotactile feedback on postural sway during locomotor activities. J. Neuroeng. Rehabil..

[27] Ma C.Z., Wan A.H., Wong D.W., Zheng Y.-P., Lee W.C. Improving postural control using a portable plantar pressure-based vibrotactile biofeedback system. Proceedings of the IEEE Conference on Biomedical Engineering and Sciences (IECBES).

[28] Huang Q.-H., Zheng Y.-P., Chena X., He J.F., Shi J. (2007). A system for the synchronized recording of sonomyography, electromyography and joint angle. Open Biomed. Eng. J..

[29] Shi J., Zheng Y.-P., Huang Q.-H., Chen X. (2008). Continuous monitoring of sonomyography, electromyography and torque generated by normal upper arm muscles during isometric contraction: Sonomyography assessment for arm muscles. IEEE Trans. Biomed. Eng..

[30] Zhou G.-Q., Zheng Y.-P., Zhou P. (2017). Measurement of Gender Differences of Gastrocnemius Muscle and Tendon Using Sonomyography during Calf Raises: A Pilot Study. BioMed Res. Int..

[31] Zhou G.-Q., Chan P., Zheng Y.-P. (2015). Automatic measurement of pennation angle and fascicle length of gastrocnemius muscles using real-time ultrasound imaging. Ultrasonics.

[32] Wettenschwiler P.D., Stämpfli R., Lorenzetti S., Ferguson S.J., Rossi R.M., Annaheim S. (2015). How reliable are pressure measurements with Tekscan sensors on the body surface of human subjects wearing load carriage systems?. Int. J. Ind. Ergon..

[33] Lempereur M., Brochard S., Leboeuf F., Rémy-Néris O. (2014). Validity and reliability of 3D marker based scapular motion analysis: A systematic review. J. Biomech..

[34] Barker S., Craik R., Freedman W., Herrmann N., Hillstrom H. (2006). Accuracy, reliability, and validity of a spatiotemporal gait analysis system. Med. Eng. Phys..

[35] Plewa K., Samadani A., Chau T. (2017). Comparing electro- and mechano-myographic muscle activation patterns in self-paced pediatric gait. J. Electromyogr. Kinesiol..

[36] Richards J. (2018). The Comprehensive Textbook of Clinical Biomechanics.

[37] Plewa K. (2018). Analysis and Interpretation of Lower Limb Mechanomyographic Signals in Human Gait. Ph.D. Thesis.

[38] Elhadi M.M.O., Ma C.Z., Wong D.W.C., Wan A.H.P., Lee W.C.C. (2017). Comprehensive gait analysis of healthy older adults who have undergone long-distance walking. J. Aging Phys. Act..

[39] Elhadi M.M.O., Ma C.Z.-H., Lam W.K., Lee W.C.-C. (2018). Biomechanical Approach in Facilitating Long-Distance Walking of Elderly People Using Footwear Modifications. Gait Posture.

[40] Jaskólski A., Andrzejewska R., Marusiak J., Kisiel-Sajewicz K., Jaskólska A. (2007). Similar response of agonist and antagonist muscles after eccentric exercise revealed by electromyography and mechanomyography. J. Electromyogr. Kinesiol..

